# Machine Learning for Predicting Individual Severity of Blepharospasm Using Diffusion Tensor Imaging

**DOI:** 10.3389/fnins.2021.670475

**Published:** 2021-05-13

**Authors:** Gang Liu, Yanan Gao, Ying Liu, Yaomin Guo, Zhicong Yan, Zilin Ou, Linchang Zhong, Chuanmiao Xie, Jinsheng Zeng, Weixi Zhang, Kangqiang Peng, Qingwen Lv

**Affiliations:** ^1^Department of Neurology, The First Affiliated Hospital, Sun Yat-sen University, Guangzhou, China; ^2^Guangdong Provincial Key Laboratory for Diagnosis and Treatment of Major Neurological Diseases, National Key Clinical Department and Key Discipline of Neurology, Guangzhou, China; ^3^School of Biomedical Engineering, Southern Medical University, Guangzhou, China; ^4^Department of Information, Zhujiang Hospital, Southern Medical University, Guangzhou, China; ^5^Department of Medical Imaging, Sun Yat-sen University Cancer Center, State Key Laboratory of Oncology in Southern China, Collaborative Innovation Center for Cancer Medicine, Guangzhou, China

**Keywords:** blepharospasm, fractional anisotropy, Jankovic Rating Scale, local diffusion homogeneity, machine learning

## Abstract

Accumulating diffusion tensor imaging (DTI) evidence suggests that white matter abnormalities evaluated by local diffusion homogeneity (LDH) or fractional anisotropy (FA) occur in patients with blepharospasm (BSP), both of which are significantly correlated with disease severity. However, whether the individual severity of BSP can be identified using these DTI metrics remains unknown. We aimed to investigate whether a combination of machine learning techniques and LDH or FA can accurately identify the individual severity of BSP. Forty-one patients with BSP were assessed using the Jankovic Rating Scale and DTI. The patients were assigned to non-functionally and functionally limited groups according to their Jankovic Rating Scale scores. A machine learning scheme consisting of beam search and support vector machines was designed to identify non-functionally versus functionally limited outcomes, with the input features being LDH or FA in 68 white matter regions. The proposed machine learning scheme with LDH or FA yielded an overall accuracy of 88.67 versus 85.19% in identifying non-functionally limited versus functionally limited outcomes. The scheme also identified a sensitivity of 91.40 versus 85.87% in correctly identifying functionally limited outcomes, a specificity of 83.33 versus 83.67% in accurately identifying non-functionally limited outcomes, and an area under the curve of 93.7 versus 91.3%. These findings suggest that a combination of LDH or FA measurements and a sophisticated machine learning scheme can accurately and reliably identify the individual disease severity in patients with BSP.

## Introduction

Primary blepharospasm (BSP) is the second most common primary adult-onset dystonia (Hallett et al., 2008). BSP is not only characterized by motor manifestations, including orbicularis oculi spasms, apraxia of eyelid opening, and increased blinking, but also by nonmotor aspects, including sensory symptoms, psychiatric disturbances, sleep abnormalities, and cognitive dysfunction (Defazio et al., 2017). Motor and non-motor manifestations contributing to BSP have the potential to substantially influence quality of life, and even lead to functional blindness. However, the exact etiology and pathophysiological mechanisms of BSP are not entirely clear.

Clinical evaluation of BSP involves many challenges, particularly in the severity rating (Krack and Marion, 1994). Currently, the severity of BSP is mainly assessed by various types of scales in both routine clinical practice and research settings. However, the wide use of existing severity scales is subject to criticism of varying extent (Albanese et al., 2013b; Defazio et al., 2015, 2017). In addition, in the traditional rating scale, the raters (usually doctors) are required to communicate with the patients face-to-face for a period of time, and then give the score. The reliability of the scale can be reduced not only because the raters need special training, but also due to subjective errors caused by patients and raters. In recent years, accumulating neuroimaging evidence suggests that structural magnetic resonance image (sMRI) and diffusion tensor imaging (DTI) can be applied to investigate brain structural alterations in various types of primary adult-onset dystonia, including BSP. Gray matter changes in the basal ganglia, primary sensorimotor cortex, cingulate/paracingulate cortex, and cerebellum have been found in patients with BSP (Obermann et al., 2007; Martino et al., 2011; Suzuki et al., 2011; Horovitz et al., 2012). However, the lack of any correlation between severity of BSP and gray matter changes in these areas suggest that sMRI has a limited value in evaluating the BSP severity. Nevertheless, a recent DTI study based on 31 BSP patients revealed significant fractional anisotropy (FA) decreases in the white matter of the left anterior lobe of the cerebellum, which was significantly correlated with disease severity (Yang et al., 2014). In addition, our previous study also found widespread white matter abnormalities evaluated by local diffusion homogeneity (LDH) in 29 patients with BSP, which was also significantly correlated with disease severity (Guo et al., 2020). FA, which is one of the most commonly used DTI indexes, quantifies the degree of anisotropy characterized by the random motion of water molecules preferentially directed along the axis of the major axonal pathway, which only reflects diffusion properties within the voxel (Melhem et al., 2002). LDH quantifies the overall similarity of water molecules diffusion profiles between voxels and their adjacent voxels, which is considered to reflect the local consistency of fiber orientation, density, diameter, or myelination of white matter (Gong, 2013). In general, a higher FA or LDH value represents the microstructural reorganization of brain white matter, and reduced FA or LDH indicates the microstructural disruption of neural fibers (Tang et al., 2012; Liu et al., 2017; Guo et al., 2020). These studies suggest that DTI has the potential to evaluate the individual severity of BSP, but the specific DTI markers for identifying the individual severity of BSP remain unknown.

Machine learning has advantages of flexibility and scalability relative to traditional biostatistical methods, which makes it be applicable in many clinical fields, such as diagnosis and classification, risk stratification, and survival predictions. In addition, machine learning has the ability to analyze diverse data types, such as imaging data, laboratory findings, and demographic data, and extracts features from data that humans may not be able to do (Ngiam and Khor, 2019). Moreover, machine learning enables analysis at the individual level compared with traditional biostatistical methods that compute significance and effects at the group level, which guarantees a proportion of aid in the diagnosis and treatment of individual patients in the clinical practice (Kim and Na, 2018).

The goal of this study was to construct a sophisticated machine learning model for accurately identifying the individual severity of BSP (non-functionally and functionally limited) based on diffusion metrics (FA and LDH) in 68 white matter regions. Another purpose of the study was to identify brain structural correlates of possible pathophysiological mechanisms of BSP according to the frequency of each feature in FA or LDH feature subsets. We chose this method because the frequency of each feature may implicitly indicate a correlation between a specific brain structure and the severity of BSP. In other words, the diffusion indices of the brain structures with the highest frequency may be highly associated with the severity of BSP and may be efficient neuroimaging biomarkers of the severity of BSP. We hypothesized that FA or LDH, combined with an appropriate machine learning scheme, can accurately identify the individual severity of BSP.

## Materials and Methods

### Participants

This study was approved by the First Affiliated Hospital of Sun Yat-sen University Clinical Research Review Board ([2020]323). Oral and written informed consent was attained from each participant either personally or by proxy. Patients were consecutively recruited from our outpatient clinic for movement disorders between April 2019 and July 2020. Inclusion criteria included: (1) patients aged 18–75 years; (2) patients diagnosed with BSP according to the published standard criteria (Albanese et al., 2013a) by a senior neurologist (G Liu) with long-standing experience in movement disorders; and (3) recruited patients treated with botulinum toxin (BoNT) at the end of their treatment cycle, at least 3 months post-injection. Exclusion criteria were the following: (1) patients with metallic medical implants that were contraindicated for magnetic resonance image (MRI); (2) patients with traumatic brain injury, stroke, epilepsy, Parkinson’s disease, Alzheimer’s disease, psychiatric diseases, or evidence of possible anxiety [Hamilton Anxiety Scale (HAMA) score > 14; Hamilton, 1959); (3) patients with a history of drug or alcohol abuse; and (4) patients with abnormal findings on conventional MRI and known causes of secondary dystonia. We also included age- and gender-matched healthy controls. All subjects were right-handed.

### Clinical Assessments

The most widely used severity scale specifically developed for BSP is the Jankovic Rating Scale (JRS), which includes both severity subscale (0 = None, 1 = Increased blinking only with external stimulus, 2 = Mild but spontaneous eyelid fluttering, but not functionally disabling, 3 = Moderate spasm, mildly incapacitating, and 4 = Severe, incapacitating spasm including eyelid and other facial muscles) and frequency subscale (0 = None, 1 = Slightly increased blinking frequency, 2 = Eyelid fluttering shaking lasting less than 1 second, 3 = Orbicularis oculi muscle spasm lasting more than 1 second with eyes opening more than 50% of awake time, and 4 = Functionally “blind”) (Jankovic and Orman, 1987; Defazio et al., 2017). Therefore, the severity of BSP was assessed immediately before MRI scanning based on a JRS score of 0–4. The JRS scores were used to assign patients to a non-functionally limited group (spasm intensity scores <3 and/or spasm frequency scores <4) or a functionally limited group (spasm intensity scores ≥3 and spasm frequency scores = 4). The JRS was performed by a trained neurologist (YM Guo) who was blind to the clinical information of patients with BSP.

### MRI Data Acquisition

Magnetic resonance image data for all subjects were acquired using a 3T scanner (Tim Trio; Siemens, Erlangen, Germany) with a 12-channel head coil. DTI data were acquired using a spin-echo, echo planar imaging sequence in 50 axial planes with 64 non-collinear directions (b = 1,000 s/mm^2^), and a non-diffusion-weighted volume (b = 0 s/mm^2^). Scan parameters were as follows: flip angle = 90°, echo time = 91 ms, repetition time = 7,000 ms; 128 × 128 matrix dimensions; 2 mm × 2 mm × 3 mm voxel size; and 256 mm × 256 mm of view.

### DTI Data Preprocessing and Feature Extraction

We employed the DTI pipeline software PANDA (Pipeline for Analyzing Brain Diffusion Images)^1^ to perform DTI data preprocessing (Cui et al., 2013). More detailed information about the DTI data preprocessing can be found in our previous studies (Liu et al., 2017; Guo et al., 2020). A total of 68 white matter regions were extracted from the ICBM-DTI-81 white-matter labels atlas (rICBM_DTI_81_WMPM_90p_FMRIB58; Oishi et al., 2008) and then we calculated the averaged FA and LDH values of each region using the normalized diffusion parameter maps. More details about each structure can be found in Supplementary Table 1.

### Feature Normalization

Since each feature has a different value range, the role of features with higher values in the comprehensive analysis is highlighted while the role of features with lower values is relatively weakened. The zero-mean normalization method was used to normalize the data for the purpose of improving the reliability and accuracy of the model. Specifically, the standardization of each feature was calculated as below

$$x_{ij}^{{}^{*}}=\frac{x_{ij}-\mu \left(x_{j}\right)}{\sigma \left(x_{j}\right)},$$

where *x*_*ij*_ denotes the value of the *j*th feature of the *i*th patient, μ(*x*_*j*_) denotes the mean value of the *j*th feature of all patients, σ(*x*_*j*_) denotes the standard deviation of the *j*th feature of all patients, and $x_{ij}^{*}$ denotes the normalized feature value. The same normalization procedure was used in both training and test samples to make the experiments more precise.

### Feature Selection and Classifier

**FIGURE 1:**
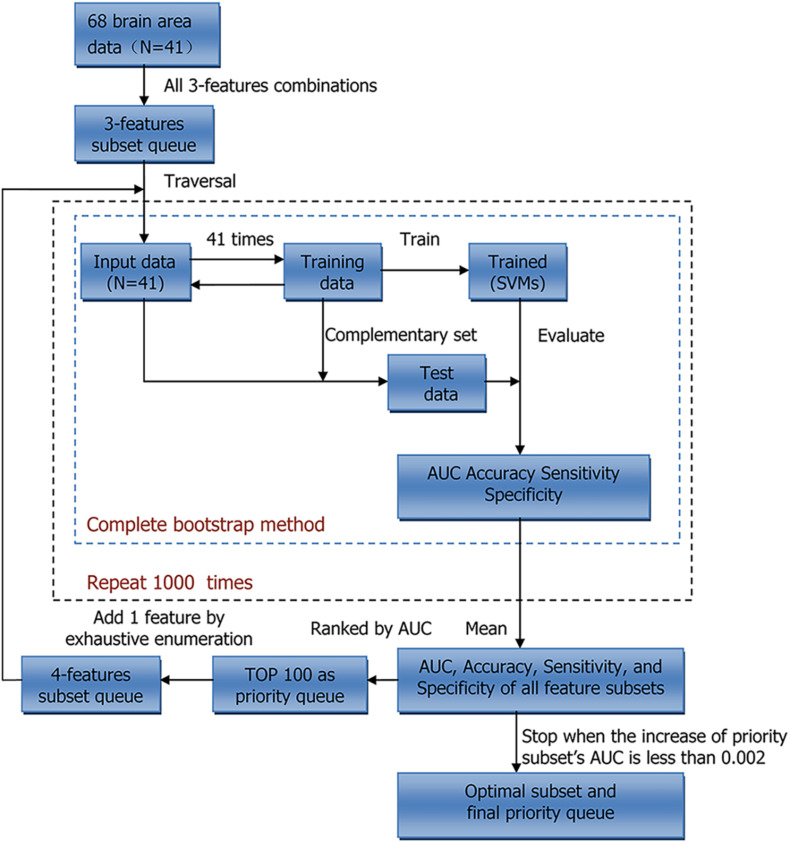
Workflow of Beam search. N indicates sample size. AUC, area under curve; SVMs, support vector machines.

We conducted two sets of experiments using LDH and FA, respectively. Principal component analysis (PCA), ReliefF, and beam search (BS) were used to reduce feature dimensions. PCA is a reduction method that uses orthogonal transformation to map original features to a set of new features with smaller dimensions (Sirovich and Kirby, 1987). ReliefF, a feature weighting algorithm, assigns different weights to each feature according to the relevance of each feature and category (Robnik-Sikonja and Kononenko, 2003). Both PCA and ReliefF are fast and commonly used, but their effect is usually worse than that of a complete search. As such we also used BS to select features as accurately as possible (Sabuncuoglu and Bayiz, 1999). The entire procedure for BS is shown in Figure 1; within each loop, each feature subset was used as input data for a model to classify the samples. In order to select the stable feature subsets, the average area under curve (AUC) of a 1,000 times bootstrap validation was used as the evaluation index of each feature subset, and all feature subsets were ranked by AUC. First, we performed three feature combinations on 68 features to obtain a feature subset queue with a size of 50,116. Then, we traversed this queue to classify the samples and took the feature subset with the highest AUC as the priority feature subset, and similarly, the feature subsets with the top 100 AUC as the priority queue. Finally, we exhaustively added a feature to these subsets in the priority queue to form a new feature subset queue. This process was repeated until the AUC of the priority feature subset of the next loop no longer increased significantly, i.e., the increase of the AUC was less than 0.002, to get the optimal feature subset and the final priority queue. In addition, classification was based on the mapping relationship between the feature and label. The frequency of each feature may implicitly indicate the correlation between the corresponding brain structure and severity of BSP. We also calculated the frequency of each feature in the final priority queue.

In classifying non-functionally and functionally limited groups, support vector machines (SVMs) were used to construct the classifier, which is a small sample learning method (Hsu and Lin, 2002). AUC, accuracy, sensitivity, and specificity were used to evaluate the performance of the classifier.

### Bootstrap Validation and Stratified Five-Fold Cross-Validation

Due to the small sample size, we conducted two different validation methods, bootstrap validation and stratified five-fold cross-validation, to improve the robustness of the observation results.

Bootstrap validation is based on bootstrap sampling, which is useful for small data sets and the condition of hard dividing into training and test sets. Within each iteration, one sample was randomly selected from 41 samples and put into the training set, then put back into the sample set. After being repeated 41 times, a training set containing 41 samples was obtained, and the samples that were not selected would be used as the test set. The training set was used to train the model, and correspondingly, the test set was used to evaluate the model’s performance. The procedure above was repeated 1,000 times to calculate the average results, which was regarded as the bootstrap validation result of the model.

**FIGURE 2:**
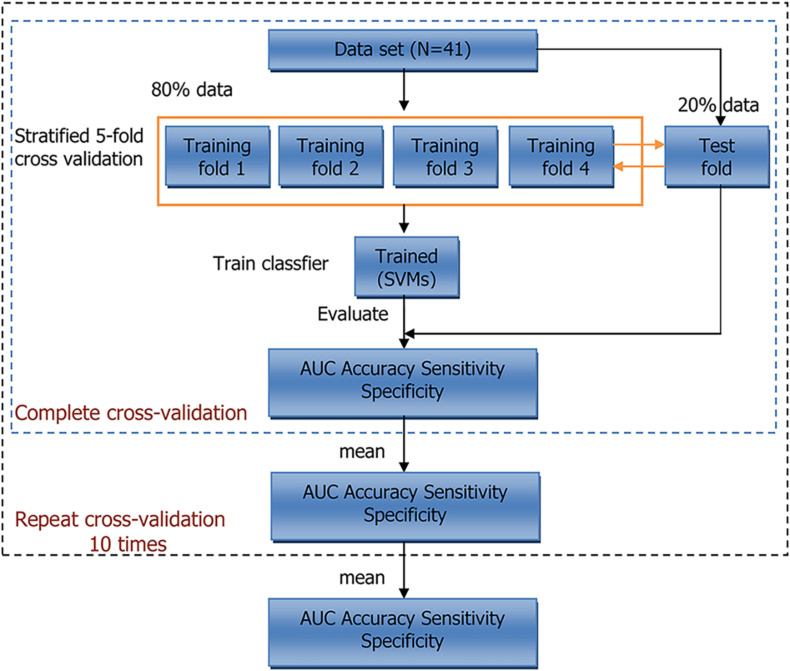
Workflow of 5-fold cross-validation. N indicates sample size. AUC, area under curve; SVMs, support vector machines.

However, the bootstrap method changes the distribution of the initial data set, which introduces estimation bias. To account for this, we also performed five-fold cross-validation. The entire training process is shown in Figure 2. Within each iteration, one-fold was used as the test set to evaluate the trained model, and the remaining folds were used to train model. This procedure was repeated five times, and the average result of five-folds was regarded as cross-validation result of the model. At last, we calculated the average results of 10 repeats of cross-validation. Scikit-learn (version: 0.23.1) software package was used in our experiments (Pedregosa et al., 2011).

### Statistical Analysis

In analyzing demographic information, clinical characteristics, and behavioral test scores, categorical data were compared between groups using Pearson chi-square or Fisher exact tests (when the expected number was ≤5). Parametric data were compared using the one-way ANOVA after normality testing by the Shapiro–Wilk test, and nonparametric data were compared using the Mann–Whitney U test. All analyses were performed using SPSS 16.0 for Windows software (SPSS Inc., Chicago, IL, United States) and statistical significance was set at *P* < 0.05.

## Results

### Participant Characteristics and Behavioral Evaluations

Three patients were excluded from analyses due to early termination of scanning (*n* = 1) and stroke lesions (*n* = 2). This resulted in a final study sample of 41 patients (26 women and 15 men; median age, 53 years). The demographic information, behavioral test scores, and clinical characteristics for both groups are shown in Table 1. The functionally limited group had higher JRS scores than the non-functionally limited group (*P* < 0.001). No significant differences in age, gender, educational level, duration, and BoNT duration were found between the groups.

**TABLE 1:** Subjects demographics and clinical assessments.

	Non-functionally limited group (*n* = 12)	Functionally limited group (*n* = 29)	Healthy control group (*n* = 29)
Median age, years (range)	52 (28–74)	54 (38–75)	54 (37–75)
Female/male ratio	1	2.22	1.9
Education, years (range)	12 (9–16)	12 (0–16)	–
Median JRS (range)	5 (2–5)	6 (4–8)*	–
Median duration, years (range)	5.5 (1–12)	8 (1–25)	–
Median BoNT duration, years (range)	2 (0–7)	2 (0–20)	–

*P < 0.05, compared to the non-functionally group. BoNT, botulinum neurotoxin; JRS, Jankovic rating scale.

### Classification Performance

When using BS to select features of LDH, the AUC of the priority feature subset gradually increased as the number of features increased. When 10 features were selected, the AUC increased only slightly (Supplementary Table 2). Therefore, 10 features were selected as an optimal subset of LDH. The corresponding brain structures were the corticospinal tract (CT), left inferior cerebellar peduncle (ICP), left middle frontal blade (MFB), left parieto-temporal blade, right posterior limb of internal capsule (PLIC), right superior parietal blade (SPB), right CT, left precentral blade (PB), right ICP, and right tapetum. The frequency of each brain structure in the final priority queue is shown in Supplementary Table 3. As shown in Table 2, the 10 features with the highest frequencies were the optimal subset we selected. No significant differences in the LDH values in these brain regions between groups (non-functionally limited group versus functionally limited group, non-functionally limited group versus healthy control group, and functionally limited group versus healthy control group) were observed (Supplementary Table 4). In addition, we performed the same operation on the FA (Supplementary Table 5), and the corresponding frequencies of brain structures are shown in Supplementary Table 6. The optimal FA feature subset we selected contained 3 features, including the right posterior corona radiata, left CT, and left ICP, which fit the most frequently appearing features in the final priority queue perfectly (Table 2). Moreover, no marked differences in the FA values in these brain structures between groups were observed (Supplementary Table 4).

**TABLE 2:** Full name and frequency of each brain structure in the optimal feature subset of LDH and FA.

Feature	Index	Full name	Location	Frequency
LDH	8	Corticospinal tract	Left	1
	12	Inferior cerebellar peduncle	Left	1
	54	Middle frontal blade	Left	1
	64	Parieto-temporal blade	Left	1
	19	Posterior limb of internal capsule	Right	0.92
	61	Superior parietal blade	Right	0.84
	7	Corticospinal tract	Right	0.8
	58	Pre-central blade	Left	0.7
	11	Inferior cerebellar peduncle	Right	0.58
	49	Tapetum	Right	0.53
FA	27	Posterior corona radiata	Right	0.53
	8	Corticospinal tract	Left	0.31
	12	Inferior cerebellar peduncle	Left	0.25

*FA, fractional anisotropy; LDH, local diffusion homogeneity.*

The results of all classification experiments are shown in Table 3. The machine learning scheme we proposed, with BS, SVMs, and multiple (10×) stratified five-fold cross-validation, achieved an AUC of 0.937 and an overall accuracy of 88.67% when using LDH features. Furthermore, it had an accuracy of 91.40% in correctly identifying functionally limited BSP patients (i.e., sensitivity) and an accuracy of 83.33% in identifying non-functionally limited BSP patients (i.e., specificity). When FA features were used as input data, the model also presented a good classification performance. More specifically, the model achieved an AUC of 0.913 and an overall accuracy of 85.19%. In addition, it had a sensitivity of 85.87% and a specificity of 83.67%.

**TABLE 3:** A summary of all classifications results (mean and deviation).

Feature	Validation	Feature selection	AUC	Accuracy (%)	Sensitivity (%)	Specificity (%)
LDH	Bootstrap	None	0.506 ± 0.156	66.45 ± 9.04	91.36 ± 10.55	9.82 ± 17.96
		PCA	0.458 ± 0.143	65.09 ± 8.83	92.63 ± 9.92	3.43 ± 13.05
		ReliefF	0.639 ± 0.149	69.56 ± 9.98	93.41 ± 8.93	15.59 ± 21.02
		**BS**	**0.895 ± 0.097**	**85.06 ± 8.71**	**94.27 ± 7.58**	**66.09 ± 25.00**
	Stratified 5-fold cross validation	None	0.438 ± 0.066	68.36 ± 3.71	91.93 ± 3.69	12.00 ± 6.35
		PCA	0.360 ± 0.073	65.64 ± 4.51	85.73 ± 5.40	18.00 ± 5.41
		ReliefF	0.474 ± 0.110	72.53 ± 4.20	94.13 ± 3.80	21.00 ± 6.84
		**BS**	**0.937 ± 0.041**	**88.67 ± 3.40**	**91.40 ± 3.69**	**83.33 ± 8.43**
FA	Bootstrap	None	0.422 ± 0.141	59.73 ± 9.35	82.01 ± 14.45	10.44 ± 17.05
		PCA	0.483 ± 0.156	65.28 ± 8.91	90.62 ± 9.32	6.39 ± 15.88
		ReliefF	0.597 ± 0.136	65.55 ± 8.70	88.24 ± 11.25	15.22 ± 19.55
		**BS**	**0.886 ± 0.085**	**82.06 ± 9.42**	**85.01 ± 11.22**	**76.93 ± 23.12**
	Stratified 5-fold cross validation	None	0.603 ± 0.103	57.33 ± 4.33	78.07 ± 5.23	7.67 ± 6.51
		PCA	0.534 ± 0.072	51.97 ± 5.49	70.20 ± 7.11	7.67 ± 4.73
		ReliefF	0.472 ± 0.083	63.97 ± 2.52	85.47 ± 3.12	12.0 ± 5.42
		**BS**	**0.913 ± 0.040**	**85.19 ± 3.71**	**85.87 ± 4.05**	**83.67 ± 7.52**

*Values expressed as mean ± deviation. Bold indicates the best performance in different settings. None indicates no feature selection. AUC, area under curve; BS, beam search; FA, fractional anisotropy; LDH, local diffusion homogeneity; PCA, principal component analysis.*

As shown in Table 3, when using LDH features, no matter which validation approach we choose, the performance of the feature subset we selected by BS was better. Specifically, when using cross-validation, the feature subset we selected increased the model’s AUC from 0.438 to 0.937 and the accuracy from 68.36 to 88.67%. However, in the same settings, ReliefF only increased the accuracy of the model from 68.36 to 72.53%, while PCA reduced the accuracy from 68.36 to 65.64%. We also observed similar results with the bootstrap validation; the feature subset selected by BS outperformed PCA or ReliefF. The superiority of our proposed “BS + SVMs” scheme was confirmed by such results.

## Discussion

In this study, we found that a combination of diffusion metrics, particularly LDH, and sophisticated machine learning techniques can accurately predict the severity of BSP. In addition, we identified brain structures that may be highly associated with the severity of BSP and may be efficient neuroimaging biomarkers for the severity of BSP.

We found that although both the classification accuracy of LDH and FA are satisfactory, the combinations of brain regions with the best classification performance for LDH and FA are not exactly the same. In contrast to the FA feature subset, the LDH feature subset included more widespread white matter regions. This difference is accordance with previous studies on LDH (Gong, 2013; Liu et al., 2016, 2017; Liang et al., 2019), which suggested that LDH and FA have different sensitivities to specific white matter microstructural properties under the same pathological conditions, and that LDH is complementary to the conventional diffusion markers as a novel inter-voxel diffusion measure.

We reported the frequency of each brain structure in the priority queues in the final feature subsets of LDH and FA, respectively. The frequency may implicitly represent a correlation between the specific brain structure and the severity of BSP. Although no significant differences in the diffusion metric values in the brain regions selected in our classification model between groups were found in this study, the advantages of flexibility and scalability compared with traditional biostatistical methods (Ngiam and Khor, 2019) has been demonstrated by machine learning. Moreover, machine learning has the greater ability to detect potentially distributed brain features that may more effectively characterize diseases compared with traditional biostatistical methods (Ung et al., 2014). Therefore, the 10 brain structures with the highest frequency in Supplementary Table 3 and the 3 brain structures with the highest frequency in Supplementary Table 6 (the subsets of LDH and FA features we selected) may be highly correlated with the disease severity, which further supports the important role of these brain structures in the pathophysiology of BSP. The CT, PB, PLIC, and SPB are the important components of the corticosubcortical sensorimotor networks. These findings are consistent with a previous study that demonstrated anatomical abnormalities in the corticosubcortical sensorimotor networks in focal dystonia (Delmaire et al., 2009). It has been reported that gray matter is altered in the sensorimotor cortex, basal ganglia, and cerebellum in BSP patients. These results indicate that focal dystonia is associated with abnormal anatomical connectivity of the corticosubcortical sensorimotor areas and highlight findings of the role played by sensorimotor structures and their connections in the pathophysiologic mechanisms of the disease.

The ICP is the main spinocerebellar pathway that connects the spinal cord and cerebellum. The dorsal spinocerebellar tract, a major feedback pathway, transmits sensory signals derived from movement generated by the precentral gyrus (Grimaldi and Manto, 2012). Accumulating evidence indicates that the dorsal spinocerebellar tract exhibits major connectivity with the anterior lobe of the cerebellum (Jang and Kwon, 2014, 2016). The anterior lobe of the cerebellum engages in conveying peripheral afferent signals to motor-oriented commands in the sensorimotor cortex (Ben Taib et al., 2005). A recent DTI study based on 31 BSP patients revealed significant FA decreases in the white matter of the left anterior lobe of the cerebellum, which was significantly correlated with disease severity (Yang et al., 2014). Structural and functional neuroimaging studies also showed that the cerebellum is an important contributor to the regional anomalous network model of BSP (Peller et al., 2006; Obermann et al., 2007). Our current findings indirectly support the notion that BSP may result from the abnormalities of a sensorimotor network involving the cerebellum, especially the anterior lobe of the cerebellum.

The MFB is involved in the inhibitory control of involuntary movements along with other subregions of the prefrontal cortex (Leung and Cai, 2007). A transcranial magnetic stimulation study confirmed that the cortical center of upper facial movement, including blinking, is not mainly located in facial motor cortex, but rather in the mesial frontal region (Sohn et al., 2004). A sMRI study of patients with BSP found gray matter volume changes in the right middle frontal cortex. These results suggest that in addition to the corticosubcortical areas primarily involved in sensory processing and motor control, other brain regions pertaining to the prefrontal cortex may also engage in the mechanisms underlying motor features of BSP.

Several limitations in the present study should be discussed. Only 41 subjects were used in our study, such a small sample size makes building a regression model to predict individual JRS score of patients with BSP based on DTI parameters combined with machine learning algorithms impossible. However, findings of this study preliminarily suggest that the diffusion metrics (LDH or FA) may have potential value in identifying the individual severity of BSP, which may promote future studies with larger sample sizes. In addition, the sex ratio and disease duration were different between the groups; although the potential influences of these differences on our conclusion remain unclear, these associations should be considered. Additionally, the potential influences of BoNT on DTI metrics also should be taken in consideration. Finally, our research continues on the theoretical basis of model establishment; however, the effectiveness of the model has not been further verified on large clinical samples. At present, our model was not combined with other computer technology and hardware to generate a runnable platform or application program; therefore, it has limited application in clinical practice.

## Summary

In summary, we show that a combination of LDH or FA measurements with a sophisticated machine learning scheme can accurately and reliably identify the individual disease severity in patients with BSP (non-functionally limited versus functionally limited), suggesting that DTI parameters may be of clinical value in assessing and following the individual severity of BSP. In addition, our current findings highlight an important role of corticosubcortical sensorimotor networks and other brain regions pertaining to the prefrontal cortex in the pathophysiological mechanisms underlying the motor features of BSP.

## Data Availability Statement

The datasets for this study are not publicly available because of participant privacy. Requests to access the datasets should be directed to GL.

## Ethics Statement

The study involving human participants was reviewed and approved by the First Affiliated Hospital of Sun Yat-sen University Clinical Research Review Board ([2020]323). Oral and written informed consent was attained from each participant either personally or by proxy.

## Author Contributions

GL conceived the research project and wrote and edited the manuscript. YGa designed the statistical analysis, performed the experimental work, and wrote the first draft of the manuscript. YL performed the experimental work, selected the patients, and wrote the first draft of the manuscript. YGu, ZY, ZO, and WZ assisted in selecting patients and summarized the clinical tables. CX and LZ performed the experimental work and assisted in the statistical analysis. JZ assisted in designing the experimental work. KP assisted in designing experimental work and reviewed the manuscript. QL reviewed the statistical analysis and the manuscript. All authors contributed to the article and approved the submitted version.

## Conflict of Interest

The authors declare that the research was conducted in the absence of any commercial or financial relationships that could be construed as a potential conflict of interest.

## Footnotes

1 http://www.nitrc.org/projects/panda/
