# Supplementary material for: Machine Learning for Predicting Individual Severity of Blepharospasm Using Diffusion Tensor Imaging
Source: Front Neurosci. 2021 May 13;15:670475. doi: 10.3389/fnins.2021.670475 (PMC8155629; doi:10.3389/fnins.2021.670475)
Supplement: Supplementary file 1 [file Data_Sheet_1.pdf]

**SUPPLEMENTARY MATERIAL**

**Machine learning for predicting individual severity of blepharospasm using diffusion tensor imaging**

Gang Liu[1,2†], Yanan Gao[3,4†], Ying Liu[1,2†], Yaomin Guo[1,2], Zhicong Yan[1,2], Zilin Ou[1,2], Linchang Zhong[5], Chuanmiao Xie[5], Jinsheng Zeng[1,2], Weixi Zhang[1,2], Kangqiang Peng[5]*, Qingwen Lv[4]*

[1]Department of Neurology, The First Affiliated Hospital, Sun Yat-sen University, Guangzhou, China, [2]Guangdong Provincial Key Laboratory for Diagnosis and Treatment of Major Neurological Diseases, National Key Clinical Department and Key Discipline of Neurology, Guangzhou, China, [3]School of Biomedical Engineering, Southern Medical University, Guangzhou, China, [4]Department of Information, Zhujiang Hospital, Southern Medical University, Guangzhou, China, [5]Department of Medical Imaging, Sun Yat-sen University Cancer Center, State Key Laboratory of Oncology in Southern China, Collaborative Innovation Center for Cancer Medicine, Guangzhou, China

**Supplementary Tables**

**Table 1** Full name of 68 white matter regions

| Index | Full name | Location |
|---|---|---|
| 1 | Middle cerebellar peduncle | |
| 2 | Pontine crossing tract (a part of middle cerebellar peduncle) | |
| 3 | Genu of corpus callosum | |
| 4 | Body of corpus callosum | |
| 5 | Splenium of corpus callosum | |
| 6 | Fornix (column and body of fornix) | |
| 7 | Corticospinal tract | Right |
| 8 | Corticospinal tract | Left |
| 9 | Medial lemniscus | Right |
| 10 | Medial lemniscus | Left |
| 11 | Inferior cerebellar peduncle | Right |
| 12 | Inferior cerebellar peduncle | Left |
| 13 | Superior cerebellar peduncle | Right |
| 14 | Superior cerebellar peduncle | Left |
| 15 | Cerebral peduncle | Right |
| 16 | Cerebral peduncle | Left |
| 17 | Anterior limb of internal capsule | Right |
| 18 | Anterior limb of internal capsule | Left |
| 19 | Posterior limb of internal capsule | Right |
| 20 | Posterior limb of internal capsule | Left |
| 21 | Retrolenticular part of internal capsule | Right |
| 22 | Retrolenticular part of internal capsule | Left |
| 23 | Anterior corona radiata | Right |
| 24 | Anterior corona radiata | Left |
| 25 | Superior corona radiata | Right |
| 26 | Superior corona radiata | Left |
| 27 | Posterior corona radiata | Right |
| 28 | Posterior corona radiata | Left |
| 29 | Posterior thalamic radiation (include optic radiation) | Right |
| 30 | Posterior thalamic radiation (include optic radiation) | Left |
| 31 | Sagittal stratum (include inferior longitudinal fasciculus and inferior fronto-occipital fasciculus) | Right |
| 32 | Sagittal stratum (include inferior longitudinal fasciculus and inferior fronto-occipital fasciculus) | Left |
| 33 | External capsule | Right |
| 34 | External capsule | Left |
| 35 | Cingulum (cingulate gyrus) | Right |
| 36 | Cingulum (cingulate gyrus) | Left |

| | | |
|---|---|---|
| 37 | Cingulum (hippocampus) | Right |
| 38 | Cingulum (hippocampus) | Left |
| 39 | Fornix (cres) / Stria terminalis (can not be resolved with current resolution) | Right |
| 40 | Fornix (cres) / Stria terminalis (can not be resolved with current resolution) | Left |
| 41 | Superior longitudinal fasciculus | Right |
| 42 | Superior longitudinal fasciculus | Left |
| 43 | Superior fronto-occipital fasciculus (could be a part of anterior internal capsule) | Right |
| 44 | Superior fronto-occipital fasciculus (could be a part of anterior internal capsule) | Left |
| 45 | Inferior fronto-occipital fasciculus | Right |
| 46 | Inferior fronto-occipital fasciculus | Left |
| 47 | Uncinate fasciculus | Right |
| 48 | Uncinate fasciculus | Left |
| 49 | Tapetum | Right |
| 50 | Tapetum | Left |
| 51 | Superior frontal blade | Right |
| 52 | Superior frontal blade | Left |
| 53 | Middle frontal blade | Right |
| 54 | Middle frontal blade | Left |
| 55 | Inferior frontal blade | Right |
| 56 | Inferior frontal blade | Left |
| 57 | Pre-central blade | Right |
| 58 | Pre-central blade | Left |
| 59 | Post-central blade | Right |
| 60 | Post-central blade | Left |
| 61 | Superior parietal blade | Right |
| 62 | Superior parietal blade | Left |
| 63 | Parieto-temporal blade | Right |
| 64 | Parieto-temporal blade | Left |
| 65 | Temporal blade | Right |
| 66 | Temporal blade | Left |
| 67 | Occipital blade | Right |
| 68 | Occipital blade | Left |

**Table 2** A summary of priority feature subsets based on LDH by beam search (mean ± deviation)

| Feature subset | AUC | Accuracy/% | Sensitivity/% | Specificity/% |
|---|---|---|---|---|
| 1-feature | 0.676 ± 0.160 | 63.52 ± 11.63 | 65.92 ± 16.69 | 59.63 ± 26.14 |
| 2- features | 0.781 ± 0.151 | 77.81 ± 10.51 | 84.36 ± 12.50 | 63.54 ± 24.64 |
| 3- features | 0.847 ± 0.111 | 75.26 ± 9.89 | 81.42 ± 12.47 | 63.00 ± 25.13 |
| 4- features | 0.845 ± 0.125 | 78.09 ± 10.88 | 85.69 ± 11.91 | 63.73 ± 26.59 |
| 5- features | 0.863 ± 0.098 | 80.23 ± 10.21 | 89.74 ± 10.36 | 60.98 ± 26.18 |
| 6- features | 0.881 ± 0.097 | 81.97 ± 10.40 | 89.85 ± 10.22 | 67.46 ± 26.35 |
| 7- features | 0.885 ± 0.096 | 82.74 ± 8.82 | 91.31 ± 9.66 | 65.56 ± 25.60 |
| 8- features | 0.889 ± 0.097 | 83.87 ± 9.10 | 91.45 ± 8.89 | 68.48 ± 26.01 |
| 9- features | 0.893 ± 0.095 | 84.92 ± 8.75 | 95.14 ± 7.24 | 63.45 ± 25.00 |
| **10- features** | **0.895 ± 0.097** | **85.06 ± 8.72** | **94.27 ± 7.58** | **66.09 ± 25.00** |

Values expressed as mean ± deviation. Bold indicates the best performance of the LDH feature subset. AUC, area under curve; LDH, local diffusion homogeneity.

**Table 3** Full name and frequency of each brain structure in the final priority queue of LDH

| Index | Full name | Location | Frequency |
|---|---|---|---|
| **8** | **Corticospinal tract** | **Left** | **1** |
| **12** | **Inferior cerebellar peduncle** | **Left** | **1** |
| **54** | **Middle frontal blade** | **Left** | **1** |
| **64** | **Parieto-temporal blade** | **Left** | **1** |
| **19** | **Posterior limb of internal capsule** | **Right** | **0.92** |
| **61** | **Superior parietal blade** | **Right** | **0.84** |
| **7** | **Corticospinal tract** | **Right** | **0.8** |
| **58** | **Pre-central blade** | **Left** | **0.7** |
| **11** | **Inferior cerebellar peduncle** | **Right** | **0.58** |
| **49** | **Tapetum** | **Right** | **0.53** |
| 50 | Tapetum | Left | 0.38 |
| 27 | Posterior corona radiata | Right | 0.36 |
| 26 | Superior corona radiata | Left | 0.18 |
| 13 | Superior cerebellar peduncle | Right | 0.14 |
| 52 | Superior frontal blade | Left | 0.14 |
| 68 | Occipital blade | Left | 0.13 |
| 4 | Body of corpus callosum | | 0.07 |
| 42 | Superior longitudinal fasciculus | Left | 0.06 |
| 57 | Pre-central blade | Right | 0.06 |
| 56 | Inferior frontal blade | Left | 0.04 |
| 59 | Post-central blade | Right | 0.03 |
| 2 | Pontine crossing tract (a part of middle cerebellar peduncle) | | 0.01 |
| 25 | Superior corona radiata | Right | 0.01 |
| 28 | Posterior corona radiata | Left | 0.01 |
| 53 | Middle frontal blade | Right | 0.01 |

Bold indicates 10 LDH features with the highest frequency were the optimal subset of LDH. LDH, local diffusion homogeneity.

**Table 4** The LDH and FA values of selected regions in non-functionally limited, functionally limited, and HC groups

| Feature | Anatomical regions | Location | Non-functionally limited group (mean ± SD) | Functionally limited group (mean ± SD) | HC group (mean ± SD) |
|---|---|---|---|---|---|
| LDH | Corticospinal tract | Left | 0.43 ± 0.04 | 0.42 ± 0.04 | 0.43 ± 0.04 |
| | Inferior cerebellar peduncle | Left | 0.40 ± 0.03 | 0.42 ± 0.03 | 0.41 ± 0.04 |
| | Middle frontal blade | Left | 0.49 ± 0.06 | 0.51 ± 0.05 | 0.49 ± 0.06 |
| | Parieto-temporal blade | Left | 0.47 ± 0.05 | 0.51 ± 0.04 | 0.51 ± 0.05 |
| | Posterior limb of internal capsule | Right | 0.71 ± 0.03 | 0.70 ± 0.03 | 0.70 ± 0.03 |
| | Superior parietal blade | Right | 0.51 ± 0.01 | 0.49 ± 0.04 | 0.59 ± 0.07 |
| | Corticospinal tract | Right | 0.43 ± 0.05 | 0.42 ± 0.03 | 0.42 ± 0.03 |
| | Pre-central blade | Left | 0.54 ± 0.05 | 0.55 ± 0.03 | 0.60 ± 0.04 |
| | Inferior cerebellar peduncle | Right | 0.39 ± 0.03 | 0.40 ± 0.05 | 0.40 ± 0.04 |
| | Tapetum | Right | 0.49 ± 0.09 | 0.44 ± 0.04 | 0.44 ± 0.05 |
| FA | Posterior corona radiata | Right | 0.40 ± 0.01 | 0.41 ± 0.02 | 0.41 ± 0.03 |
| | Corticospinal tract | Left | 0.51 ± 0.02 | 0.49 ± 0.03 | 0.50 ± 0.03 |
| | Inferior cerebellar peduncle | Left | 0.43 ± 0.02 | 0.43 ± 0.02 | 0.43 ± 0.02 |

FA, fractional anisotropy; HC, healthy controls; LDH, local diffusion homogeneity; SD, standard deviation.

**Table 5** A summary of priority feature subset based on FA by beam search (mean and deviation)

| Feature subset | AUC | Accuracy/% | Sensitivity/% | Specificity/% |
|---|---|---|---|---|
| 1-feature | 0.741 ± 0.141 | 67.39 ± 10.90 | 67.52 ± 16.27 | 68.48 ± 25.80 |
| 2- features | 0.756 ± 0.146 | 68.29 ± 11.50 | 75.62 ± 14.54 | 53.02 ± 27.95 |
| **3- features** | **0.886 ± 0.085** | **82.06 ± 9.42** | **85.01 ± 11.22** | **76.93 ± 23.12** |
| 4- features | 0.885 ± 0.095 | 83.17 ± 9.50 | 87.65 ± 11.20 | 74.91 ± 23.31 |
| 5- features | 0.869 ± 0.104 | 79.49 ± 10.17 | 87.96 ± 10.46 | 62.35 ± 26.12 |
| 6- features | 0.868 ± 0.103 | 81.63 ± 10.98 | 90.48 ± 10.30 | 63.55 ± 27.60 |
| 7- features | 0.849 ± 0.122 | 80.68 ± 10.54 | 89.34 ± 10.19 | 61.73 ± 26.13 |
| 8- features | 0.845 ± 0.124 | 80.05 ± 10.56 | 89.91 ± 10.33 | 59.84 ± 26.70 |
| 9- features | 0.849 ± 0.124 | 81.57 ± 10.68 | 89.40 ± 9.40 | 67.20 ± 28.42 |
| 10- features | 0.850 ± 0.118 | 81.97 ± 10.35 | 90.36 ± 9.82 | 66.32 ± 26.37 |

Values expressed as mean ± deviation. Bold indicates the best performance of the FA feature subset. AUC, area under curve; FA, fractional anisotropy.

**Table 6** Full name and frequency of each brain structure in the final priority queue of FA

| Index | Full name | Location | Frequency |
|---|---|---|---|
| **27** | **Posterior corona radiata** | **Right** | **0.53** |
| **8** | **Corticospinal tract** | **Left** | **0.31** |
| **12** | **Inferior cerebellar peduncle** | **Left** | **0.25** |
| 57 | Pre-central blade | Right | 0.25 |
| 30 | Posterior thalamic radiation (include optic radiation) | Left | 0.1 |
| 11 | Inferior cerebellar peduncle | Right | 0.09 |
| 25 | Superior corona radiata | Right | 0.09 |
| 32 | Sagittal stratum (include inferior longitudinal fasciculus and inferior fronto-occipital fasciculus) | Left | 0.09 |
| 41 | Superior longitudinal fasciculus | Right | 0.09 |
| 20 | Posterior limb of internal capsule | Left | 0.06 |
| 42 | Superior longitudinal fasciculus | Left | 0.06 |
| 60 | Post-central blade | Left | 0.06 |
| 19 | Posterior limb of internal capsule | Right | 0.05 |
| 24 | Anterior corona radiata | Left | 0.05 |
| 29 | Posterior thalamic radiation (include optic radiation) | Right | 0.05 |
| 31 | Sagittal stratum (include inferior longitudinal fasciculus and inferior fronto-occipital fasciculus) | Right | 0.05 |
| 37 | Cingulum (hippocampus) | Right | 0.05 |
| 48 | Uncinate fasciculus | Left | 0.05 |
| 49 | Tapetum | Right | 0.05 |
| 52 | Superior frontal blade | Left | 0.04 |
| 58 | Pre-central blade | Left | 0.04 |
| 62 | Superior parietal blade | Left | 0.04 |
| 3 | Genu of corpus callosum | | 0.03 |
| 6 | Fornix (column and body of fornix) | | 0.03 |
| 10 | Medial lemniscus | Left | 0.03 |
| 15 | Cerebral peduncle | Right | 0.03 |
| 18 | Anterior limb of internal capsule | Left | 0.03 |
| 22 | Retrolenticular part of internal capsule | Left | 0.03 |
| 38 | Cingulum (hippocampus) | Left | 0.03 |
| 1 | Middle cerebellar peduncle | | 0.02 |
| 14 | Superior cerebellar peduncle | Left | 0.02 |
| 28 | Posterior corona radiata | Left | 0.02 |
| 35 | Cingulum (cingulate gyrus) | Right | 0.02 |
| 39 | Fornix (cres) / Stria terminalis (can not be resolved with current resolution) | Right | 0.02 |
| 50 | Tapetum | Left | 0.02 |

| | | | |
|---|---|---|---|
| 51 | Superior frontal blade | Right | 0.02 |
| 65 | Temporal blade | Right | 0.02 |
| 66 | Temporal blade | Left | 0.02 |
| 5 | Splenium of corpus callosum | | 0.01 |
| 7 | Corticospinal tract | Right | 0.01 |
| 13 | Superior cerebellar peduncle | Right | 0.01 |
| 21 | Retrolenticular part of internal capsule | Right | 0.01 |
| 23 | Anterior corona radiata | Right | 0.01 |
| 34 | External capsule | Left | 0.01 |
| 36 | Cingulum (cingulate gyrus) | Left | 0.01 |
| 44 | Superior fronto-occipital fasciculus (could be a part of anterior internal capsule) | Left | 0.01 |
| 46 | Inferior fronto-occipital fasciculus | Left | 0.01 |
| 47 | Uncinate fasciculus | Right | 0.01 |
| 54 | Middle frontal blade | Left | 0.01 |
| 55 | Inferior frontal blade | Right | 0.01 |
| 59 | Post-central blade | Right | 0.01 |
| 61 | Superior parietal blade | Right | 0.01 |
| 64 | Parieto-temporal blade | Left | 0.01 |
| 67 | Occipital blade | Right | 0.01 |

Bold indicates 3 FA features with the highest frequency were the optimal subset of FA.
FA, fractional anisotropy.
